# Partial volume effect on kidney stones and lung nodules in CT imaging

**DOI:** 10.1371/journal.pone.0334597

**Published:** 2025-10-16

**Authors:** Andreas Christe, Beat Roth, Daniel Guido Fuster, Karim Shakarchi, Dionysios Drakopoulos, Elias Primetis, Georgios Delimpasis, Adrian Thomas Huber, Verena Carola Obmann, Alan Arthur Peters, Lukas Ebner, Grazia Maria Cereghetti

**Affiliations:** 1 Department of Diagnostic, Interventional, and Pediatric Radiology, Inselspital, Bern University Hospital, University of Bern, Bern, Switzerland; 2 Department of Urology, Inselspital, Bern University Hospital, University of Bern, Bern, Switzerland; 3 Department of Nephrology and Hypertension, Inselspital, Bern University Hospital, University of Bern, Bern, Switzerland; PLOS: Public Library of Science, UNITED KINGDOM OF GREAT BRITAIN AND NORTHERN IRELAND

## Abstract

**Rationale and Objectives:**

To examine the impact of the partial volume effect (PVE) on the imaging of spherical objects depending on their size, density and center voxel position.

**Materials and Methods:**

We developed an algorithm for calculating the volume of a sphere wrapped by voxels. The algorithm measured the internal volume of each voxel cut by the sphere and automatically attributed the average voxel density. The sphere volume was simulated by the sum of voxels with an average density above the Hounsfield Unit (HU) cutoff level for that object. Various sphere sizes, densities and positions in the voxel grid were examined. The two clinical settings used were nodules (0 HU) in the lung (−1000 HU) and kidney stones (1000 HU) embedded in the renal parenchyma (30 HU).

**Results:**

Small kidney stones appeared magnified by the PVE when a stone cutoff level of 130 HU was used: the smallest stone simulated with a diameter of 1.4 mm demonstrated a volume that was 231% the size of the ground truth (sphere volume as measured with the classical formula). A hypothetical stone of 10 cm would still have a PVE of 2%. The PVE did not affect lung nodules if the cutoff level for the nodule fraction was set to the exact mean of both the internal and external density (−500 HU). Lung nodules were more affected by the geometrical effect, where tiny nodules appeared smaller because of the greater curvature of smaller spheres, often cutting less than 50% of the volume of a surface voxel.

**Conclusions:**

This study highlights the potential risks associated with inaccurate raw data postprocessing of CT images with objects that are particularly sensitive to the PVE, such as kidney stones and high-density calcifications (Agatston score).

## 1. Introduction

In a computed tomography (CT) scanner, the object under examination is crossed by an X-ray beam originating from a rotating source and ending on detector elements located opposite the source. Photons that escape the scanned object are captured by the detector elements and converted into a signal. For each full rotation (360 degrees) of the X-ray source, the overall absorption, or “attenuation”, of each path crossing the object is measured [1,2]. The collected raw projections constitute a matrix of voxels whose size is limited by the size of the detector elements and the increment of the beam along the object (slice length). For each slice, assuming axial acquisition, the absorption values in the field of view (FOV) are typically distributed in a 512 × 512 matrix. The projection data are manipulated by mathematical algorithms to reconstruct a two-dimensional image of the slice on the basis of the calculated individual densities at each imaged location [3–5].

The detector elements of conventional CT scanners, the so-called energy integrating detectors, measure X-ray absorption after converting incident X-ray photons into visible light photons. Therefore, the spectral resolution is limited, and the raw signal in voxels encompassing materials with different attenuation properties, e.g., muscle and bone, is equal to the weighted density of both materials, whereas information about the density of each material is lost [6,7]. The voxels indeed have density numbers corresponding to the average density of both materials combined depending on their fractional contribution to the voxel, resulting in an artifact (PVE), i.e., an intermediate value that might be very different from the density numbers of the original materials [1,8].

The PVE ultimately affects the image resolution because of blurring at the intersection between structures with different attenuation properties, e.g., organ boundaries. Depending on the image reconstruction algorithm used and the imaged tissues and organs, this effect might be reduced, allowing some spatial information about the edges of the tissues to be obtained [9]. The PVE is more pronounced when the cross-sectional matrix is smaller (e.g., with an 80 × 80 image matrix than with a 1024 × 1024 image matrix) [2], and it is worsened by widening the detector width. In the early eighties, at the beginning of CT imaging, the PVE appeared especially along the z-axis, since the slice thickness (z-axis, 9 mm) was much larger than the axial voxel size (0.08 mm in a 512 × 512 image matrix). The most famous example of the PVE was the sternoclavicular joint projecting into the upper lungs, simulating a Pancoast tumor [1]. Since then, CT techniques have improved tremendously toward isotropic voxel (isovoxel) imaging [2]. In particular, the slice thickness could be decreased to the submillimeter range. The newest CT models even provide potential voxel edges of 0.3 mm with a 1024 × 1024 matrix [2]. Nevertheless, while advancements have been made to mitigate its impact, the PVE remains a significant challenge in CT imaging, even with the newest technology [10,11].

In CT imaging, density numbers are expressed in Hounsfield Units (HU), with the signal attenuation of water set to 0 HU and the signal of air set to −1000 HU [12]. The attenuation coefficient of other substances is calculated on the basis of these reference values, e.g., cortical bone has density values ranging from 500–900 HU [13]. Density values are represented graphically on CT images with a scale of gray shades.

The PVE is particularly prominent when a high-attenuation structure is adjacent to a low-attenuation structure with an HU threshold for visibility or volume measurement closer to the density of one of the two structures, causing the other structure to appear enlarged. Kidney stones, for example, have densities varying between 879 ± 230 HU (calcium oxalate monohydrate stones), 844 ± 346 HU (apatite stones) and 550 ± 74 HU (cystine stones) [14], whereas the surrounding normal renal parenchyma has an average density in the range of 30–50 HU (CT without contrast agent) [15]. A voxel encompassing both the renal parenchyma and a calcium oxalate monohydrate stone would be attributed a density of over 400 HU, leading to the attribution of the voxel to the stone considering the standard kidney stone density threshold of 130 HU (calcium) [16]. A similar situation is found in the assessment of the extent of coronary artery calcification by the Agatston score and therefore in the evaluation of cardiovascular risk [17,18] or when analyzing lung nodules [19–22]. This issue is particularly crucial when considering that nodule size is a key factor in determining malignancy risk, and overestimation of the nodule volume due to the PVE may lead to false-positive results in cancer diagnosis, particularly in cases of smaller lesions [23,24]. Incorrect nodule size measurement may also compromise the assessment of nodule growth, which affects the determination of malignancy risk [22]. Analogously, the PVE is crucial for determining the degree of malignancy of other types of lesions, especially small metastases, such as brain, liver, bone, and lymph node metastases [25,26]. Size misattributions caused by the PVE are therefore notable and have important implications in several key areas, such as diagnostic accuracy, treatment planning, treatment response assessment, and patient monitoring.

Although the PVE is a well-recognized artifact that interferes with the diagnostic accuracy of medical imaging, there is no specific algorithm to predict its impact on CT images. The purpose of this study was to establish an algorithm to simulate the PVE in a theoretical computed tomography imaging model and use the algorithm to quantify the extent of its impact on the size assessment of small spherical objects surrounded by a matrix with very different densities, using lung nodules and kidney stones as paradigmatic examples.

## 2. Materials and methods

### 2.1. Establishment of an algorithm to simulate the PVE of a spherical object embedded in a matrix

#### 2.1.1. Calculation of the number of voxels needed to completely cover a sphere.

**Fig 1:**
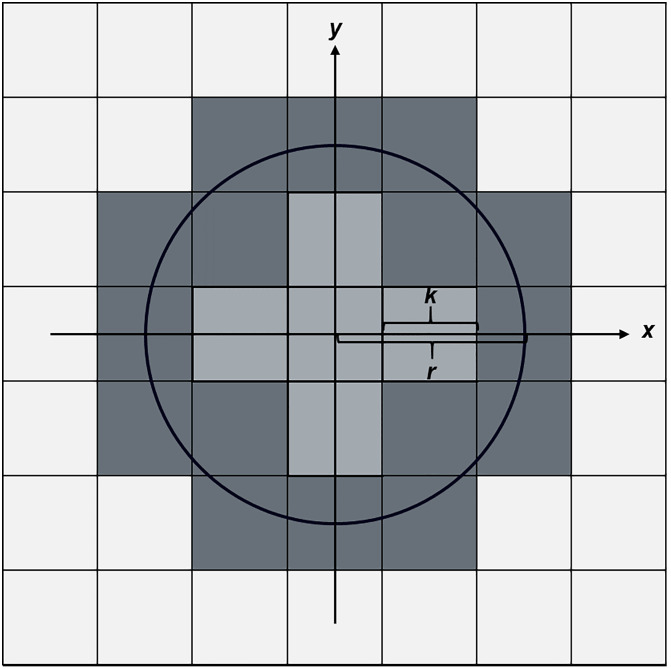
Two-dimensional cross-section of a three-dimensional sphere in the x-y plane (black circle). The center of the circle coincides with the middle point of a pixel with edge length k positioned in a grid of equivalent pixels (7x7=49 pixels). Sixteen pixels (dark gray squares) are partially included within the circle area, whereas 5 pixels are entirely included, and 28 pixels lie completely outside of the circle. In the corresponding three-dimensional space, 7 voxels lie completely inside, and 66 voxels only partially overlap with the sphere volume (considering the sphere center positioned in the middle of the central voxel).

**Fig 2:**
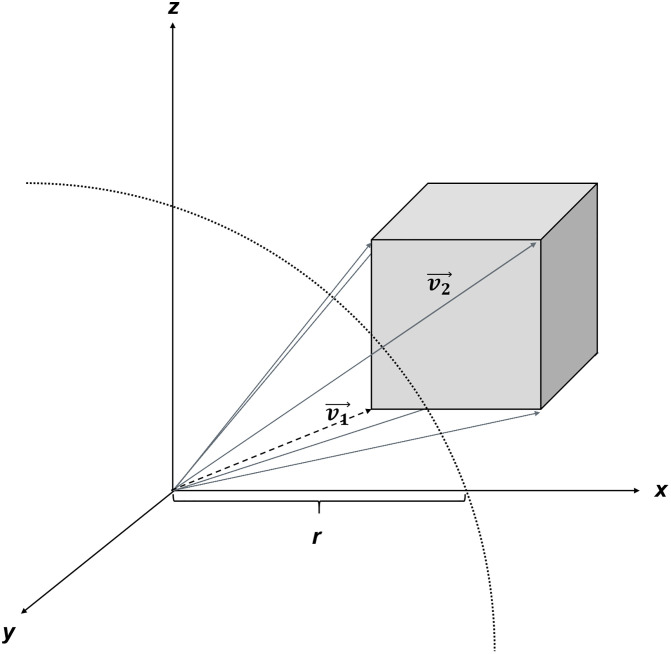
Isovoxel (gray cube) crossed by a sphere of radius r (outlined by the dotted line). Vectors (gray and dotted black arrows) originating from the sphere center define the eight voxel corners in three-dimensional space. The voxel (gray cube) is only partially included in the sphere because one vector ((v_1)^→^) is smaller (dotted black arrow) and at least one vector ((v_2)^→^) is larger (gray arrows) than the sphere radius **r.**

A three-dimensional voxel grid in an x-y-z coordinate system was used to calculate the number of voxels needed to approximate a sphere of radius r in its entirety. The sphere cross-section intersecting the sphere center was represented by a 512 × 512 virtual matrix of pixels with a voxel edge length of *k*. The sphere radius and diameter were multiples of k (Fig 1). In the 3D space, the isovoxels were defined by vectors originating in the sphere center and pointing to each of the 8 cube corners ($\vec{v}$_1_ to $\vec{v}$_8_, Fig 2). A voxel was cut by the sphere when at least one vector was smaller ($\vec{v}$_1_ dashed arrow in Fig 2) and when at least one vector was larger (e.g., $\vec{v}$_2_, one of the light gray arrows in Fig 2) than the sphere radius. If all vectors were smaller than the sphere radius, the whole voxel was contained in the sphere, whereas it was completely outside the sphere when all vectors defining its corners were longer than r.

In the simulation, the center of the coordinate system was then randomly shifted inside the center voxel volume to perform 100 simulations of the number of voxels needed to wrap the whole sphere in each position.

Python Code Summary (lung nodules; the python code used for the simulation is available in the S1 Appendix):

- **Setup**
  - Define Hounsfield Units for “inside” (HU**i**=0) and “outside” (HU**o**=-1000) the sphere.
  - Set a threshold (CUT=-500 HU) for classification of cut voxels.
- **Main Loops**:
  - Iterate over sphere radii (F) and random sphere positions within a 3D matrix.
  - For each voxel in the matrix, compute its relationship to the sphere.
- **Voxel Classification**
  - **Inside Sphere**: If all corners and the center of the voxel are within the sphere, mark it as “inside.”
  - **Outside Sphere**: If all corners and the center of the voxel are outside the sphere, mark it as “outside.”
  - **Cut by Sphere**: If some corners are inside and some are outside, the voxel is “cut.”
- **Cut Voxel Processing**
  - Use 125 evenly spaced measurement points within the voxel to determine the volume fraction inside the sphere (G).
  - Compute the voxel’s average Hounsfield Unit (HU) using G: HU=G×HU**i**+(1−G)×HU**o**
  - Classify based on HU:
    - HU>CUT: Count voxel as “inside.”
    - Otherwise: Count as “outside.”
- **Output**:
  - Track metrics such as total voxels tested, voxels inside, voxels outside, cut voxels, and their fraction inside the sphere.

The calculation of a voxel volume cut by a sphere is very complicated and time-consuming. Thus, a volume estimation using evenly distributed sample points in the voxel was run, and the volume measurement errors for each category of sampling points were simulated:

**Table:** 

Sampling Points per Voxel	Average Volume Error (%)
1	40.30
8	11.82
27	6.27
64	4.09
125	2.92
216	2.34
343	2.00
512	1.79
729	1.69
1000	1.61

The suggested 125-point method provides a reasonably accurate estimate of the cut volume, with an average volume measurement error of less than 3%. Increasing to 1000 points would further improve accuracy (~1.61% average error) but at the cost of greater computational effort and with a relatively low impact on the overall results, which would be obtained by measuring many voxels per single lung nodule or kidney stone with 100 random shifts in the center of the coordinate system (sphere).

#### 2.1.2 Estimation of the sphere‒voxel intersection volume V_cut_.

**Fig 3:**
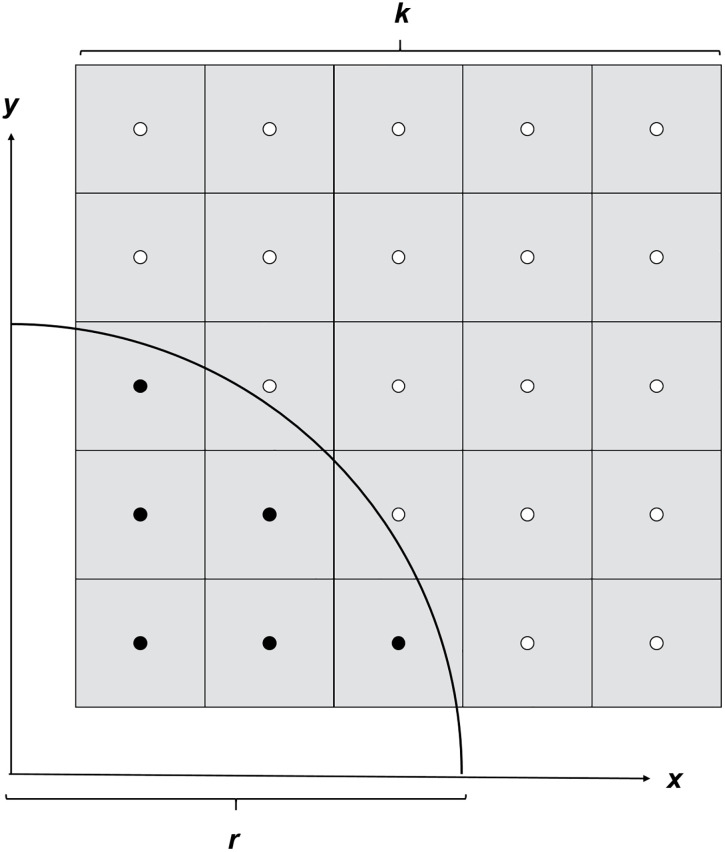
Estimation of the area of pixels cut by a circle. To estimate the area of the pixel with edge length k that is cut by a circle with radius r, a grid of 25 points was defined within the pixel. In this two-dimensional example of a voxel cross-section, 6 (vI, black dots) of 25 points lie within the circular area delineated by the black line. In the corresponding three-dimensional model of the voxel, 125 points were distributed the same way throughout the voxel to estimate the volume of the voxel effectively included in the sphere.

Because the sphere surface is bent, the section of the sphere surface intersecting the voxel is not on a plane. To outline such a curved surface and estimate the voxel volume cut by the sphere, we used a subdivision sampling method in which we fixed 125 reference points homogenously distributed within a standard voxel with an edge length *k* (Fig 3). A number of reference points exceeding 100 was aimed for and randomly set to 125. The margin of the sphere surface was traced using vectors originating from the sphere center to each of these reference points, located at 0, 1/5 and 2/5 the distance from the voxel center in each of the six directions of the x, y, z coordinate system (i.e., ± x, ± y, and ±z). The number of vectors to the 5 × 5 × 5 reference points that were shorter than the sphere radius defined the intersection volume. A large object with a radius larger than 100 times the voxel edge length would cut the voxel almost on a plane.

The sphere‒voxel intersection volume *V*_*cut*_ was calculated from the number of reference points inside the sphere (Fig 3):


$$V_{cut}=v_{I}\frac{k^{\text{3}}}{\text{1}\text{2}\text{5}}$$ (1)

*v*_*I *_= number of reference units constituting the sphere‒voxel intersection volume

Consequently, the partial sphere‒voxel intersection volume *p*_*cut*_ was given by the following equation:


$$p_{cut}=\frac{v_{I}\frac{k^{\text{3}}}{\text{1}\text{2}\text{5}}}{k^{\text{3}}}=\frac{v_{I}}{\text{1}\text{2}\text{5}}$$ (2)

*v*_*I *_= number of reference units constituting the sphere‒voxel intersection volume

#### 2.1.3 Comparison between the measured sphere volume and the real sphere volume (ground truth).

The volume of a sphere with radius *r* (*V*_*m_sphere*_) embedded in *n* voxels with an edge length *k* was calculated by adding the volume of all voxels completely inside the sphere and the sum of all sphere‒voxel intersection volumes (Equation 1):


$$V_{m\_sphere}=n_{I}k^{\text{3}}+\sum_{x=\text{1}}^{x=n_{T}}v_{I\_x}\frac{k^{\text{3}}}{\text{1}\text{2}\text{5}}$$ (3)

*n*_*I *_= number of voxels completely inside the sphere

*n*_*T*_ = number of voxels cut by the sphere

*v*_*I_x*_ = number of reference units constituting the sphere‒voxel intersection volume

in voxel *v*_*x*_

For comparison of the sphere volume value obtained from Equation (3) with the real volume (ground truth = *V*_*r_sphere*_), we used the following classical equation:


$$V_{r\_sphere}=\frac{\text{4}}{\text{3}}\pi r^{\text{3}}$$ (4)

The voxel size in CT depends on multiple parameters. For example, the average FOV in abdominal and chest CT imaging measures 35 cm, which corresponds to a voxel edge *k* of 0.68 mm in a 512 × 512 image matrix, whereas on a standard head CT with an average FOV of 20 cm, the voxel size *k* would be 0.39 mm. To account for this variability, we used for the simulation the radius unit *r*_*norm*_, corresponding to the radius size normalized to the voxel edge length *k*, instead of the effective sphere radius *r* (r can always be expressed in multiples of the edge length k (5)).


$$r_{norm}=\frac{r}{k}$$ (5)

We calculated the effective sphere volume (*V*_*r_sphere_norm*_, ground truth) with the following equation:


$$V_{r\_sphere\_norm}=\frac{\text{4}}{\text{3}}\pi {r_{norm}}^{\text{3}}$$ (6)

The simulation was performed considering a random distribution of the sphere center with respect to the voxel center. The precision of the approximation *P*_*approx*_ of the sphere volume with the method described compared with the ground truth was a function of the voxel edge *k* and the normalized sphere radius *r*_*norm*_:


$$P_{approx}=\frac{V_{m\_sphere}}{V_{r\_sphere\_norm}}=\frac{n_{I}k^{\text{3}}+\sum_{x=\text{1}}^{x=n_{T}}v_{I\_x}\frac{k^{\text{3}}}{\text{1}\text{2}\text{5}}}{\frac{\text{4}\pi {r_{norm}}^{\text{3}}}{\text{3}}}=\frac{\text{3}(n_{I}k^{\text{3}}+\sum_{x=\text{1}}^{x=n_{T}}v_{I_{x}}\frac{k^{\text{3}}}{\text{1}\text{2}\text{5}})}{\text{4}\pi {r_{norm}}^{\text{3}}}$$ (7)

*V*_*m_sphere*_* = * measured sphere volume

*V*_*r_sphere_norm*_* = * normalized, effective sphere volume

*n*_*I *_=  number of voxels completely inside the sphere

*n*_*T *_=  number of voxels cut by the sphere

*r*_*norm*_ =  normalized sphere radius

For *P*_*approx *_= 1, the simulated normalized sphere volume corresponded to the ground truth (normalized real sphere volume). For *P*_*approx *_> 1, the measured volume exceeded the real volume, and for *P*_*approx *_< 1, the volume of the real sphere was underestimated.

#### 2.1.4 Calculation of the volume of spherical objects in CT images based on voxel density cutoff values.

We applied the approximation described in sections 1–3 to calculate the PVE in the raw projection data of 2 paradigmatic clinical examples: spherical lung nodules surrounded by air and spherical kidney stones surrounded by renal parenchyma. For simplicity, we assumed the X-ray source to consist of a uniform parallel beam and the collimator, detector element and acquisition increment (pitch) to be the same size; we also assumed the same isovoxel edge size. Furthermore, we assumed zero Compton scattering and a uniform attenuation value for each material. Both kidney stones and lung nodules were assumed to be perfectly spherical.

The overall density of voxels containing two materials/tissues was obtained via the following formula:


$${HU}_{tot}=p_{\text{1}}{HU}_{I}+{(\text{1}-p}_{\text{1}}){HU}_{O}$$ (8)

*p*_*1*_* *= volume percentage of the spherical object inside the cut voxel (calculated with Equation (3))

*HU*_*I*_: density of the spherical object (“inside”)

*HU*_*O*_: density of the surrounding matrix (“outside”)

Depending on the density threshold (*HU*_*cut-off*_), voxels in virtual CT images were attributed either to the sphere or to the surrounding matrix upon calculation of *HU*_*tot*_ for each voxel wrapping the sphere.

The partial sphere‒voxel intersection volume for a voxel with *HU*_*tot *_*= HU*_*cut-off*_ was the following:


$$p_{cut-off}=\frac{{HU}_{cut-off}-{HU}_{O}}{{(HU}_{I}-{HU}_{O})}$$ (9)

**Fig 4:**
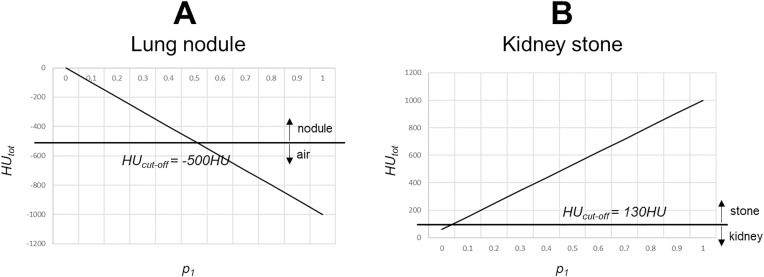
Impact of the cutoff position within the density window on the total density of the voxel. **A** – Example of lung nodules: symmetric density distribution above and below the cutoff density value of −500 HU (window −1000 HU–0 HU) with voxels belonging to the nodule when HUtot ≥ −500 HU and voxels belonging to the lung (air) if HUtot < −500 HU. **B** – Example of kidney stones: asymmetric density distribution above and below the cutoff density value of 130 HU (window 30 HU–1000 HU), with voxels belonging to the kidney stone when HUtot ≥ 130 HU and voxels belonging to the kidney if HUtot < 130 HU. The more asymmetric the density distribution is, the more predominant one object density will be.

Voxels with intersection volume *p*_*1*_ equal to or greater than *p*_*cut-off*_ were attributed to the sphere, whereas the others were attributed to the matrix (Fig 4).

The volume measured (*V*_*m_sphere_CT*_) for a sphere with radius *r* embedded in *n* voxels with edge *k* was therefore:


$$V_{m\_sphere\_CT}={n_{sphere}k}^{\text{3}}$$ (10)

$$n_{sphere}=\text{number of voxels with}{\text{HU}}_{tot}\geq {\text{HU}}_{cut-off}$$

Our calculation did not consider the uneven density distribution within the structures. The HU distribution follows a Gaussian curve; therefore, in reality, the number of voxels reaching the cutoff is not directly proportional to the average cutoff volume.

### 2.1 Use of the algorithm to simulate the PVE in clinically relevant settings (lung nodules and kidney stones)

The simulation for the volume analysis of lung nodules and kidney stones was performed with the following methods.

#### 2.1.1 Lung nodules.

The average density of the lung nodules was set to 0 HU [27] (*HU*_*I*_), and the average density of the lung (air) was set to −1000 HU (*HU*_*O*_). Therefore, the *HU*_*tot*_ of the resulting voxel was as follows:


$${HU}_{tot}=p_{nodule}(\text{0})+{(\text{1}-p}_{nodule})-\text{1}\text{0}\text{0}\text{0}$$ (11)

*p*_*nodule*_ = partial nodule‒lung (air) intersection volume for the voxel

A cutoff density of −500 HU was used for the identification of nodules (Fig 4A). The volume of a lung nodule measured from our simulation corresponded to the sum of the volumes of voxels with *HU*_*tot *_≥ −500 HU.


$$V_{m}=n_{I}k^{\text{3}}$$ (12)

*n*_*I *_= number of voxels with *HU*_*tot *_≥ −*500 HU*

In this case, the threshold level was set in the middle of the density window for the two adjacent materials (symmetric density distributions above and below the cutoff density value, Fig 4A).


$$-\text{5}\text{0}\text{0}={-\text{1}\text{0}\text{0}\text{0}p}_{nodule\_cut-off}$$

$$p_{nodule\_cut-off}=\frac{\text{5}\text{0}\text{0}}{\text{1}\text{0}\text{0}\text{0}}=\text{0}.\text{5}$$ (13)

If a nodule occupied 50% or more of the voxel, the voxel was considered part of the nodule.

#### 2.2.2 Kidney stones.

The average CT density of kidney stones was set to 1000 HU (*HU*_*I*_), whereas the surrounding renal parenchyma, ureter wall or urine was assumed to have an average density equal to 30 HU (*HU*_*O*_). Therefore, the *HU*_*tot*_ of the resulting voxel was as follows:


$${HU}_{tot}=p_{stone}\text{1}\text{0}\text{0}\text{0}+{(\text{1}-p}_{stone})\text{3}\text{0}$$ (14)

*p*_*stone*_ = partial stone‒parenchyma (kidney) intersection volume for the voxel

The cutoff value for the differentiation of a kidney stone from the surrounding matrix was set to 130 HU, according to the threshold originally used for the assessment of coronary artery calcium by the Agatston scoring system [17,18] but also used for the isolation of kidney stones in CT images [16, 28] (Fig 4B). The volume of a kidney stone corresponded to the sum of the volumes of voxels with *HU*_*tot *_≥ 130 HU according to Equation (11), with *n*_*I*_ corresponding to the number of these voxels.

The density cutoff value led to an asymmetric distribution within the density window, as the range of *HU*_*tot*_ for the attribution of the voxel to a kidney stone was much larger than the range for attribution to the matrix.


$$\text{1}\text{3}\text{0}={\text{9}\text{7}\text{0}p}_{stone\_cut-off}+\text{3}\text{0}$$

$$p_{stone\_cut-off}=\frac{\text{1}\text{0}\text{0}}{\text{9}\text{7}\text{0}}=\text{0}.\text{1}$$ (15)

If the voxel included even only 10% kidney stone material, it was considered part of the kidney stone. Considering the high number of voxels that contained only a small portion of the kidney stone, especially for small stones, the overall stone volume was considerably enlarged compared with its real size.

## 3 Results

### 3.1 Assessment of the precision of the calculated sphere volume vs. the real sphere volume

The results of the simulation of the volume of a sphere with radius *r* performed as described in Equation (3), positioning the sphere center in a random position in the central isovoxel with edge length *k* of an isovoxel grid in an *x*, *y*, *z* coordinate system, are reported in Table 1 for different *r*/*k* ratios.

**Table 1:** Approximation of the volume of a sphere with radius r using voxels with edge length *k.*

*r/k*	*Diameter (2r) in conv CT (mm)*	*n*_*I*_	*n*_*T*_	*%V*_*r*_	*Mean V*_*cut*_	*V*_*r_sphere_norm*_	*V*_*m_sphere*_	*P*_*sim*_
**1**	1.4	0.0	20.6	491	0.20	4.2	4.2	**1.00**
**2**	2.7	7.4	76.4	228	0.34	33.5	33.5	**1.00**
**3**	4.1	46.1	170.8	151	0.39	113.1	113.1	**1.00**
**4**	5.5	141.1	302.7	113	0.42	268.1	268.1	**1.00**
**5**	6.8	317.9	473.1	90	0.43	523.6	523.6	**1.00**
**6**	8.2	601.9	680.8	75	0.44	904.8	904.8	**1.00**
**7**	9.6	1016.4	924.7	64	0.45	1436.8	1436.7	**1.00**
**8**	10.9	1589.0	1207.5	56	0.46	2144.7	2144.7	**1.00**
**9**	12.3	2343.1	1528.2	50	0.46	3053.6	3053.7	**1.00**
**10**	13.7	3305.5	1885.6	45	0.47	4188.8	4188.8	**1.00**
**20**	27.3	29861.7	7539.9	23	0.48	33510.3	33510.3	**1.00**
**30**	41.0	104797.0	16966.5	15	0.49	113097.2	113097.3	**1.00**
**40**	54.7	253245.0	30163.3	11	0.49	268082.3	268082.6	**1.00**
**50**	68.4	500341.8	47126.5	9	0.49	523598.3	523599.1	**1.00**
**60**	82.0	871194.4	67853.0	7	0.49	904777.9	904778.2	**1.00**
**70**	95.7	1390993.2	92364.5	6	0.50	1436753.8	1436759.8	**1.00**

The approximation was performed by moving the sphere center to random positions within the central voxel. nI = number of voxels completely inside the sphere; nT = number of voxels cut by the sphere; %Vr = ratio of voxels cut to real sphere volume (geometric effect); mean Vcut = average sphere‒voxel intersection volume; Vr_sphere_norm = normalized, effective sphere volume; Vm_sphere = measured sphere volume; Psim = precision of the simulation.

**Fig 5:**
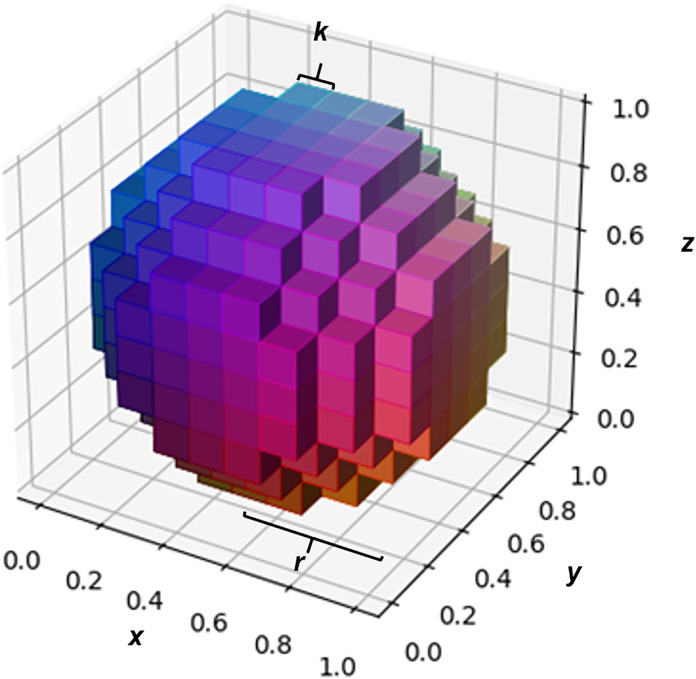
Spherical object embedded in voxels. Example of a spherical object (e.g., a spherical kidney stone) embedded in the minimum number of voxels needed to contain the whole object if its radius corresponds to four times the voxel side (r = 4k). In a 512x512 image matrix (normal abdominal CT image), this object would have a radius of 2.72 mm (diameter = 5.44 mm), with a voxel edge measuring 0.68 mm.

**Fig 6:**
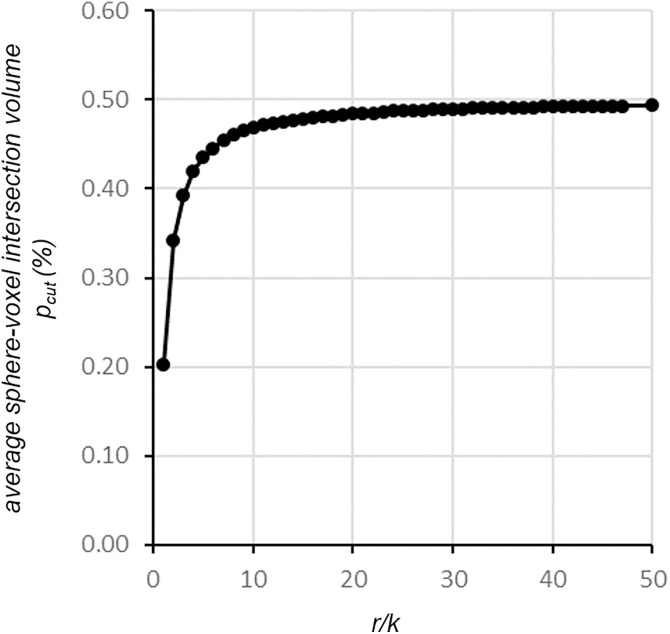
Geometrical shrinkage observed at smaller r/k ratios. The reduction in the average sphere‒voxel intersection volume (y axis) at lower r/k ratios is due to the bowed intersection surface of the sphere; k = pixel edge length, r = sphere radius.

If the sphere radius and the voxel edge were the same size (*r* = *k*, i.e., *r*_*norm*_ = 1), with a random distribution of the sphere center in the central voxel, on average, only 0.01 voxels were completely inside the sphere, whereas 20.6 voxels were cut by the sphere (492% of the real sphere volume). The partial sphere‒voxel intersection volume *p*_*cut*_ (Equation (2)) was only 20.3% on average in this case because of the high curvature of the small sphere. Doubling the sphere radius length (*r* = *2k*) increased *p*_*cut*_ to 34.2%, with 76.4 voxels cut (228% of the real sphere volume). Fig 5 shows the rendering of a sphere wrapped by voxels with edges corresponding to 1/4 of the sphere radius (*r* = 4*k*). At a radius length 20 times the voxel edge, *p*_*cut*_ (48%) was already close to the plateau value of 50% expected for this simulation, with 7540 voxels cut (22.5% of the real sphere volume). At a radius length 32 times the voxel edge, the voxel edge (corresponding to a sphere diameter of 16k) *p*_*cut*_ increased to 49% and reached a plateau for r > 70k (sphere diameter of 35k) (Table 1 and Fig 6).

The precision of the simulation *P*_*sim*_, i.e., the average ratio *P*_*approx*_ (Equation (7)) between the approximated sphere volume (*V*_*m_sphere*_, Equation (3)) and the ground truth (effective, normalized sphere volume *V*_*r_sphere_norm*_, Equation (6)) is 1 for each sphere size. At smaller sphere levels, the PVE is counteracted by the geometrical effect, where the strongly bowed intersection often cuts less than 50% of the volume of a surface voxel.

**Fig 7:**
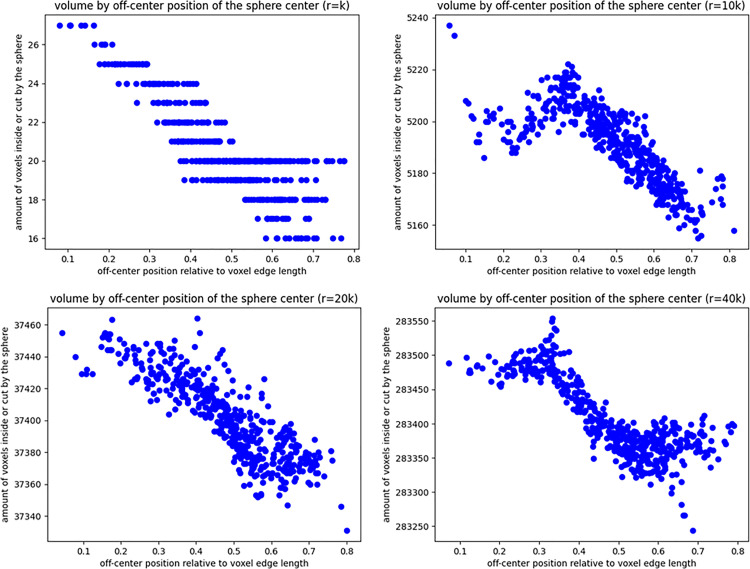
Plot of the number of voxels with edge length k crossed by a sphere with radius r at different sphere center positions with respect to the central voxel. The plots show single assessments for each position of the sphere within the central voxel for 4 different r/k ratios (r = 0k, r = 10k, r = 20k, and r = 40k). As the r/k ratio increases, the difference in the number of voxels crossed by the sphere as the sphere center moves away from the central voxel center becomes negligible.

Off-center positioning leads to smaller volume measurements, especially for small objects (Fig 7). For *r = k*, the sphere occupies 27 voxels when the sphere center is close to the voxel center, whereas when the sphere center is decentralized to the extremes of the voxel edges, only 16–20 voxels are crossed by the sphere (26–40% difference). However, the position of larger spheres does not impact the number of voxels crossed by the sphere as much. At r = 40k, the number of voxels crossed by the sphere changes by approximately 300 units of 283500 (~1‰) when the sphere center moves away from the voxel center.

### 3.2. PVE assessment for lung nodules

The results of the simulation of the volume of a spherical lung nodule, taking into consideration the threshold of −500 HU for the attribution of a voxel to the nodule, are reported in Table 2.

**Table 2:** Lung nodules surrounded by air.

*r/k*	*Diameter (2r) in conv CT (mm)*	*n*_*I*_	*n*_*T*_	*Mean V*_*cut*_	*V*_*r_sphere_norm*_	*V*_*m_sphere*_	*P*_*sim*_
**1**	1.4	0.0	20.6	0.20	4.2	3.1	**0.75**
**2**	2.7	7.4	76.4	0.34	33.5	31.2	**0.93**
**3**	4.1	46.1	170.8	0.39	113.1	109.4	**0.97**
**4**	5.5	141.1	302.7	0.42	268.1	263.9	**0.98**
**5**	6.8	317.9	473.1	0.43	523.6	518.3	**0.99**
**6**	8.2	601.9	680.8	0.44	904.8	899.9	**0.99**
**7**	9.6	1016.4	924.7	0.45	1436.8	1430.9	**1.00**
**8**	10.9	1589.0	1207.5	0.46	2144.7	2138.6	**1.00**
**9**	12.3	2343.1	1528.2	0.46	3053.6	3046.2	**1.00**
**10**	13.7	3305.5	1885.6	0.47	4188.8	4181.8	**1.00**
**20**	27.3	29861.7	7539.9	0.48	33510.3	33501.0	**1.00**
**30**	41.0	104797.0	16966.5	0.49	113097.2	113084.0	**1.00**
**40**	54.7	253245.0	30163.3	0.49	268082.3	268067.5	**1.00**
**50**	68.4	500341.8	47126.5	0.49	523598.3	523583.5	**1.00**
**60**	82.0	871194.4	67853.0	0.49	904777.9	904764.7	**1.00**
**70**	95.7	1390993.2	92364.5	0.50	1436753.8	1436740.2	**1.00**

The volume of the lung nodule, as measured on the basis of the average voxel density, is compared with the volume calculated in the simulation. The PVE disappears at small lung nodule sizes (column Psim, ratio between simulated volume and volume based on the average voxel density). nI = number of voxels completely inside the sphere; nT = number of voxels cut by the sphere; mean Vcut = average sphere‒voxel intersection volume; Vr_sphere_norm = normalized, effective sphere volume; Vm_sphere = measured sphere volume; Psim = precision of the simulation (geometrical effect).

The simulation shows that the geometrical effect of small spheres with the subsequent loss of voxels to air can be reduced to only −1% with this approximation method by a nodule radius corresponding to 5 voxels, which corresponds to a nodule diameter of 6.8 mm in an average chest CT scan with a FOV of 35 cm and an image matrix of 512 × 512 (Table 2). The shrinkage becomes negligible (−0.2%) when *r* is 10*k* (nodule diameter in conventional chest CT = 1.37 cm) and disappears from r = 17*k* (nodule diameter in conventional chest CT = 2.32 cm).

### 3.3. PVE assessment for kidney stones

The results of the simulation for kidney stones, taking into consideration the threshold of 130 HU [16,18] for the attribution of a voxel to the kidney stone, are reported in Table 3.

**Table 3:** Kidney stones surrounded by renal parenchyma/fat/urine.

*r/k*	*Diameter (2r) in conv CT (mm)*	*n*_*I*_	*n*_*T*_	*Mean V*_*cut*_	*V*_*r_sphere_norm*_	*V*_*m_sphere*_	*P*_*sim*_
**1**	1.4	0.0	20.6	0.20	4.2	9.7	**2.31**
**2**	2.7	7.4	76.4	0.34	33.5	54.7	**1.63**
**3**	4.1	46.1	170.8	0.39	113.1	159.6	**1.41**
**4**	5.5	141.1	302.7	0.42	268.1	348.4	**1.30**
**5**	6.8	317.9	473.1	0.43	523.6	649.8	**1.24**
**6**	8.2	601.9	680.8	0.44	904.8	1085.7	**1.20**
**7**	9.6	1016.4	924.7	0.45	1436.8	1681.0	**1.17**
**8**	10.9	1589.0	1207.5	0.46	2144.7	2460.9	**1.15**
**9**	12.3	2343.1	1528.2	0.46	3053.6	3451.5	**1.13**
**10**	13.7	3305.5	1885.6	0.47	4188.8	4679.7	**1.12**
**20**	27.3	29861.7	7539.9	0.48	33510.3	35459.9	**1.06**
**30**	41.0	104797.0	16966.5	0.49	113097.2	117455.2	**1.04**
**40**	54.7	253245.0	30163.3	0.49	268082.3	275822.9	**1.03**
**50**	68.4	500341.8	47126.5	0.49	523598.3	535682.2	**1.02**
**60**	82.0	871194.4	67853.0	0.49	904777.9	922137.4	**1.02**
**70**	95.7	1390993.2	92364.5	0.50	1436753.8	1460395.4	**1.02**

The volume of the kidney stone, as measured on the basis of the average voxel density, is compared with the volume calculated in the simulation. The volume measurement error is more difficult to eliminate than in the case of lung nodules, and it is still present even at kidney stone lengths above the real size (95.7 mm) (column Psim, ratio between simulated volume and volume based on the average voxel density). nI = number of voxels completely inside the sphere; nT = number of voxels cut by the sphere; mean Vcut = average sphere‒voxel intersection volume; Vr_sphere_norm = normalized, effective sphere volume; Vm_sphere = measured sphere volume; Psim = precision of the simulation (partial volume effect).

The simulation shows that the volume measurement is difficult to eliminate. For *r* = 5*k* (stone diameter in conventional abdominal CT = 6.8 mm), the precision still reaches +24%, and it is still +11.7% when *r* = 10*k* (stone diameter in conventional abdominal CT = 1.37 cm). The measurement error falls below 5% only at stone diameters >3 cm and is never lower than 2%, even when considering stone diameters in the range of 10 cm, which are nonetheless extremely uncommon (Table 3).

## 4. Discussion

In this study, we created an algorithm for simulating the volume of a spherical object wrapped in a three-dimensional voxel grid, moving its center at random positions with respect to the center of the voxel in the middle of the coordinate system. As depicted in Fig 6, the volume of the sphere‒voxel intersection divided by the voxel volume was expected to approach a plateau of 50% as the sphere radius increased with respect to the voxel edge (r>>k). For a sphere radius 70 times the voxel edge size, the intersection should lie almost on a plane, and the intersection volume should be equal to 50% on average, whereas due to the high curvature of the intersection surface in the case of a smaller sphere, the average intersection volume is reduced (e.g., 20.3% for *r = k, geometrical effect*). This effect is neutralized by the greater number of voxels cut by small spheres than by larger spheres:

The smallest examined lung nodule (r = k) cuts more voxels (20.6), but in return, the average cut voxel volume is only 20%, which is far from the 50% partial intersection volume with larger spheres (geometrical effect). On the one hand, the resulting classic volume measurement is accurate (the number of voxels cut multiplied by the average volume cut); on the other hand, the nodule volume assessed by the sum of all voxels with a density above the cutoff level is not accurate, since only 20% of the voxel volume equals the internal density of the lung nodule and 80% of the voxel volume has the external density of air. These voxels reach an average density of −800 HU, which is below the nodule cutoff level of −500 HU and will therefore be lost to the air fraction (HU effect). The combination of the geometrical and HU effects leads to an underestimation of the volume for small spheres.

The impact of the sphere position in the simulation on the number of voxels crossed by the sphere also appeared to decrease with increasing sphere radius/voxel size ratios (Fig 7). As shown in the plots in Fig 7, the position of the sphere center with respect to the central voxel plays a relevant role for smaller spheres (*r ~ k*), with up to 40% more voxels crossed by the sphere when its center is located in proximity to the middle voxel center, whereas it was negligible at r>>k (~1‰ for *r = 40k*).

We used the algorithm to evaluate the PVE under the following two frequent clinical conditions:

a) Lung nodules surrounded by air: this condition is representative of situations in which the density cutoff (threshold) for differentiating the sphere (nodule) from the surrounding matrix (lung) lies in the middle of the density window between the average densities of both materials (symmetric volume threshold level); the cutoff density for the identification of lung nodules (average density: 0 HU) in the lung (average density: −1000 HU) is indeed −500 HU.
b) Kidney stones surrounded by renal parenchyma: this condition is representative of situations in which the density cutoff for differentiating the sphere (stone) from the surrounding matrix (kidney) lies away from the middle of the density window between the average densities of both materials (asymmetric volume threshold level); the cutoff density for the identification of kidney stones (average density: 1000 HU) in the kidney (average density: 30 HU) is indeed 130 HU.

The results obtained with our algorithm show that it is possible to almost completely eliminate the PVE only when the cutoff density is in the middle of the density window (i.e., the cutoff value is close to the average of the densities of the two materials, as in situation a). The voxel matrix performed less well when the threshold density was close to one extremity of the density window, especially at wide window widths, as in situation b (asymmetric volume threshold level). With a threshold close to the density of the matrix, the size of the object is magnified (positive PVE). The geometrical effect (Fig 6) somewhat counteracted the size overestimation of the PVE in the simulations.

The PVE might be worsened by windowing if the resulting window width further sharpens the asymmetric distribution of the densities of the different materials around the cutoff level, amplifying the PVE. Considering the average kidney stone density of 1000 HU and the density of the adjacent tissues (renal parenchyma, ureter wall or urine, 0–60 HU), the classical diagnostic window for urologic CT reading (window level 50, width 450 HU) strongly emphasizes the PVE. A kidney stone with a diameter of two voxels would indeed appear to occupy 27 voxels instead of 4 voxels. On the other hand, size estimation or CT volumetry of lung nodules with the default settings of the reading system/software is accurate, especially for larger nodules. However, assessing lung nodules with a soft tissue window (window center = 50 HU) results in apparent nodule shrinkage and should therefore be avoided.

According to the outcome of our simulation in these two typical settings, assessing vessel calcifications with the Agatston score should also be performed with some precautions. The Agatston score is used especially for the diagnostic evaluation of coronary artery calcifications [17,18]. The cutoff density for calcifications is set to 130 HU [17,18]. Calcifications are evaluated based on a density factor, which depends on the greatest attenuation in the calcified area. If the density peak is in the range of 130–199 HU, the density factor is 1; it becomes 2 if the attenuation peak is in the range of 200–299 HU, 3 if the peak is between 300 and 399 HU, and 4 if the peak is larger than 400 HU. The Agatston score, or “volume”, is calculated by multiplying the density factor by the surface of the calcified area [18]. For correct calculation of the Agatston score, it is therefore fundamental that the size of the calcified area delineated on the image is as close as possible to the real size, particularly for greater density factors. Indeed, according to our results, this is true for calcifications whose densities *d*_*calc*_ lie within the following window range:


$$d_{calc}=\text{1}\text{3}\text{0}HU\pm (\text{1}\text{3}\text{0}HU-{HU}_{matrix})$$ (15)

*HU*_*matrix*_ = density of the material surrounding the calcification

**Fig 8:**
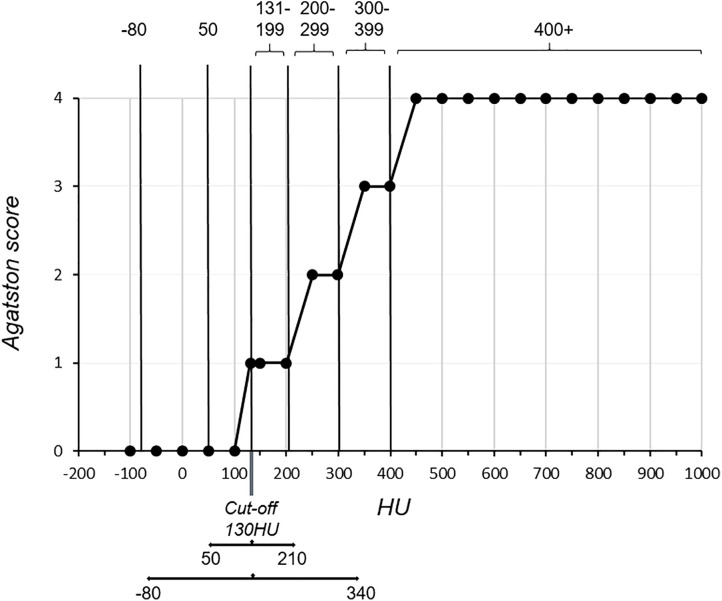
Volumetric assessment of calcifications by the Agatston score. The plot shows the Agatston score scaling and the density window limits for calcifications in blood vessels (average blood vessel density = 50 HU), as well as calcifications in pericardial fat (average pericardial fat density = −80 HU). For calcifications with densities greater than 210 HU in blood vessels and 340 HU in pericardial fat, the PVE increasingly affects the accuracy of the assessment.

The density of the vessel wall, blood or heart muscle that typically surrounds these calcifications is 50 HU on average (*HU*_*matrix*_), and the volume of only calcifications with attenuation levels in the range of 210 HU would be accurate (Fig 8). This means that for density factors greater than 1–2, the Agatston score is overestimated, and the greater the score is, the smaller the degree of calcification. In contrast, calcifications embedded in pericardial fat (*HU*_*matrix*_ corresponding to −80 HU) are better estimated because of the larger window width, as shown in Fig 8. Calcifications with density factors of 1–3 and, to some extent, also 4, are considered to more or less approximate the ground truth, particularly in cases of larger calcifications.

Considering that the amount of calcium in calcifications correlates not only with increasing density but also with increasing cardiovascular risk, the risk of a calcification with a high calcium content might be overrated. Some hard plaques with a very high calcium content have attenuation levels similar to those of kidney stones (1000 HU), and the volume assessment of these plaques is consequently very inaccurate, as they appear larger than they actually are. Even though overrating the risk might be preferable to underrating it, this would still affect the prognosis and thus also therapeutic decisions. The assessment of kidney stones (average density = 1000 HU) by the Agatston score is also discouraged because of its high degree of inaccuracy.

Our data emphasize the urgency of establishing correction factors for assessment of the Agatston score when dealing with highly calcified/high-density structures.

Our approach has several limitations. First, the simplified setting used for the simulation in the clinical cases analyzed leads to an incomplete characterization of the extent of the PVE in real case scenarios. In particular, we did not consider the physical properties of the object (e.g., the effect of Compton scattering on the signal, especially at higher tube voltages, or the mostly inhomogeneous density distribution within the material) or possible concomitant imaging artifacts or alternative scanner characteristics (e.g., fan or cone beam geometry, image magnification, etc.).

Furthermore, our simulation has the disadvantage of being restricted to the study of raw data only, i.e., it does not consider any convolution or reconstruction postprocessing, which is part of the standard image reconstruction process in CT imaging [3,4,29]. Depending on the purpose of the postprocessing algorithm (e.g., sharpening of bone edges vs. reducing noise in soft tissues), tissue edges are modified [3,5,29], which, in cases of lung nodules or kidney stones, might ultimately result in a reduction or increase in size, as previously described for some reconstruction algorithms [30–32]. The deforming impact of each algorithm used on small objects, such as those described in this study, deserves further investigation, especially if the size of such objects is relevant, e.g., for diagnostic purposes or for preoperative evaluations, because the distortions caused by the PVE characterized in this study could even be amplified by inappropriate postprocessing. Suitable corrective factors, or guidelines providing the best reconstruction algorithm to use, are needed to guarantee the minimization of the PVE, increase diagnostic performance, and support optimal therapeutic decisions. For example, kidney stones up to a threshold size do not undergo surgical treatment because they are often released spontaneously [33]. Inaccurate coronary artery calcium scoring performed to measure the coronary atherosclerotic plaque burden may lead to an incorrect prediction of cardiovascular disease risk [17,34]. The incorrect size estimation of lung nodules may also contaminate the prognostic outcome and lead to incorrect treatment choices [21,35,36].

Our study is also limited to conventional, energy integrating detector CT imaging and does not review the impact of very recent innovative CT technology with a photon counting detector (PCD), which was introduced into the clinical routine in 2021 [37–39], on the PVE. This new detector type (made of semiconductor materials such as cadmium telluride, cadmium-zinc telluride or silicon) allows the direct conversion of X-ray photons into current pulses, increasing both spatial and spectral resolution and drastically reducing the PVE [40]. This technology is intended to progressively replace conventional CT imaging technology and thus reduce the burden of the PVE in radiology.

## 5. Conclusion

Our simulations reveal the extent to which sizing highly contrasted small objects with CT imaging (especially using smaller matrix systems) is corrupted by the PVE, particularly when the spherical object analyzed is small compared with the voxel size and when the threshold density of the spherical object is not in a region close to the average attenuation values of the object and the matrix surrounding it but in the range of the matrix (positive PVE) or the object (negative PVE) density. For small spherical objects, we observe a geometrical effect (due to the spherical nature of the cut surface within each voxel) and an HU effect, which together counteract the PVE. Nevertheless, the PVE remains a challenge in CT imaging, as it impairs diagnostic accuracy and consequently treatment management. This work underlines the importance of finding suitable downstream correction factors for the assessment of CT images of structures that are particularly sensitive to the PVE. These factors would enhance image quality, improve quantitative accuracy and allow for better lesion detection. Overall, this would result in more effective treatment planning.

## Supporting information

S1 Appendix Python code (voxels cut by sphere). (DOCX)

## References

[1] Souza A, Udupa JK, Saha PK. Volume rendering in the presence of partial volume effects. IEEE Trans Med Imaging. 2005;24(2):223–35. doi: 10.1109/tmi.2004.840295 15707248

[2] National Research Council (US), Institute of Medicine (US). X-ray computed tomography. In: Committee on the Mathematics and Physics of Emerging Dynamic Biomedical Imaging, editor. Mathematics and physics of emerging biomedical imaging. Washington (DC): National Academies Press (US); 1996. 25121300

[3] Thibault J-B, Sauer KD, Bouman CA, Hsieh J. A three-dimensional statistical approach to improved image quality for multislice helical CT. Med Phys. 2007;34(11):4526–44. doi: 10.1118/1.2789499 18072519

[4] Kalra MK, Woisetschläger M, Dahlström N, Singh S, Digumarthy S, Do S, et al. Sinogram-affirmed iterative reconstruction of low-dose chest CT: effect on image quality and radiation dose. AJR Am J Roentgenol. 2013;201(2):W235–44. doi: 10.2214/AJR.12.9569 23883238

[5] Beister M, Kolditz D, Kalender WA. Iterative reconstruction methods in X-ray CT. Phys Med. 2012;28(2):94–108. doi: 10.1016/j.ejmp.2012.01.003 22316498

[6] Kak AC, Slaney M. Principles of computerized tomographic imaging: SIAM; 2001.

[7] Clackdoyle R, Defrise M. Tomographic Reconstruction in the 21st Century. IEEE Signal Process Mag. 2010;27(4):60–80. doi: 10.1109/msp.2010.936743

[8] Pullan BR, Ritchings RT, Isherwood I. Accuracy and meaning of computed tomography attenuation values. In: Newton TH, Potts DG, editors. Technical Aspects of Computed Tomography. St. Louis: Mosby. 1981: 3904–17.

[9] Schuijf JD, Lima JAC, Boedeker KL, Takagi H, Tanaka R, Yoshioka K, et al. CT imaging with ultra-high-resolution: Opportunities for cardiovascular imaging in clinical practice. J Cardiovasc Comput Tomogr. 2022;16(5):388–96. doi: 10.1016/j.jcct.2022.02.003 35210183

[10] Pelc NJ. Recent and future directions in CT imaging. Ann Biomed Eng. 2014;42(2):260–8. doi: 10.1007/s10439-014-0974-z 24435658 PMC3958932

[11] Singh P, Verma M. Technical challenges and benefits of photon counting detector computed tomography. Int J Community Med Public Health. 2024;11(8):3287–94. doi: 10.18203/2394-6040.ijcmph20242192

[12] Glide-Hurst C, Chen D, Zhong H, Chetty IJ. Changes realized from extended bit-depth and metal artifact reduction in CT. Med Phys. 2013;40(6):061711. doi: 10.1118/1.4805102 23718590

[13] Lim Fat D, Kennedy J, Galvin R, O’Brien F, Mc Grath F, Mullett H. The Hounsfield value for cortical bone geometry in the proximal humerus--an in vitro study. Skeletal Radiol. 2012;41(5):557–68. doi: 10.1007/s00256-011-1255-7 21932054

[14] Patel SR, Haleblian G, Zabbo A, Pareek G. Hounsfield units on computed tomography predict calcium stone subtype composition. Urol Int. 2009;83(2):175–80. doi: 10.1159/000230020 19752613

[15] Sagel SS, Stanley RJ, Levitt RG, Geisse G. Computed tomography of the kidney. Radiology. 1977;124(2):359–70. doi: 10.1148/124.2.359 327511

[16] Kaviani P, Primak A, Bizzo B, Ebrahimian S, Saini S, Dreyer KJ, et al. Performance of threshold-based stone segmentation and radiomics for determining the composition of kidney stones from single-energy CT. Jpn J Radiol. 2023;41(2):194–200. doi: 10.1007/s11604-022-01349-z 36331701

[17] Gupta A, Bera K, Kikano E, Pierce JD, Gan J, Rajdev M, et al. Coronary Artery Calcium Scoring: Current Status and Future Directions. Radiographics. 2022;42(4):947–67. doi: 10.1148/rg.210122 35657766

[18] Agatston AS, Janowitz WR, Hildner FJ, Zusmer NR, Viamonte M Jr, Detrano R. Quantification of coronary artery calcium using ultrafast computed tomography. J Am Coll Cardiol. 1990;15(4):827–32. doi: 10.1016/0735-1097(90)90282-t 2407762

[19] Devaraj A, van Ginneken B, Nair A, Baldwin D. Use of Volumetry for Lung Nodule Management: Theory and Practice. Radiology. 2017;284(3):630–44. doi: 10.1148/radiol.2017151022 28825886

[20] Han D, Heuvelmans MA, Oudkerk M. Volume versus diameter assessment of small pulmonary nodules in CT lung cancer screening. Transl Lung Cancer Res. 2017;6(1):52–61. doi: 10.21037/tlcr.2017.01.05 28331824 PMC5344834

[21] Larici AR, Farchione A, Franchi P, Ciliberto M, Cicchetti G, Calandriello L, et al. Lung nodules: size still matters. Eur Respir Rev. 2017;26(146):170025. doi: 10.1183/16000617.0025-2017 29263171 PMC9488618

[22] Petkovska I, Brown MS, Goldin JG, Kim HJ, McNitt-Gray MF, Abtin FG, et al. The effect of lung volume on nodule size on CT. Acad Radiol. 2007;14(4):476–85. doi: 10.1016/j.acra.2007.01.008 17368218 PMC2752296

[23] Causey JL, Zhang J, Ma S, Jiang B, Qualls JA, Politte DG, et al. Highly accurate model for prediction of lung nodule malignancy with CT scans. Sci Rep. 2018;8(1):9286. doi: 10.1038/s41598-018-27569-w 29915334 PMC6006355

[24] Gavrielides MA, Kinnard LM, Myers KJ, Petrick N. Noncalcified lung nodules: volumetric assessment with thoracic CT. Radiology. 2009;251(1):26–37. doi: 10.1148/radiol.2511071897 19332844 PMC2663581

[25] Alavi A, Werner TJ, Høilund-Carlsen PF, Zaidi H. Correction for Partial Volume Effect Is a Must, Not a Luxury, to Fully Exploit the Potential of Quantitative PET Imaging in Clinical Oncology. Mol Imaging Biol. 2018;20(1):1–3. doi: 10.1007/s11307-017-1146-y 29181818

[26] Freitas PS, Janicas C, Veiga J, Matos AP, Herédia V, Ramalho M. Imaging evaluation of the liver in oncology patients: A comparison of techniques. World J Hepatol. 2021;13(12):1936–55. doi: 10.4254/wjh.v13.i12.1936 35069999 PMC8727197

[27] Xu DM, van Klaveren RJ, de Bock GH, Leusveld ALM, Dorrius MD, Zhao Y, et al. Role of baseline nodule density and changes in density and nodule features in the discrimination between benign and malignant solid indeterminate pulmonary nodules. Eur J Radiol. 2009;70(3):492–8. doi: 10.1016/j.ejrad.2008.02.022 18417311

[28] Demehri S, Kalra MK, Rybicki FJ, Steigner ML, Lang MJ, Houseman EA, et al. Quantification of urinary stone volume: attenuation threshold-based CT method--a technical note. Radiology. 2011;258(3):915–22. doi: 10.1148/radiol.10100333 21339353

[29] Solomon J, Samei E. Quantum noise properties of CT images with anatomical textured backgrounds across reconstruction algorithms: FBP and SAFIRE. Med Phys. 2014;41(9):091908. doi: 10.1118/1.4893497 25186395

[30] Huang AE, Montoya JC, Shiung M, Leng S, McCollough CH. Consistency of Renal Stone Volume Measurements Across CT Scanner Model and Reconstruction Algorithm Configurations. AJR Am J Roentgenol. 2017;209(1):116–21. doi: 10.2214/AJR.16.16940 28402129 PMC5481469

[31] Jain R, Omar M, Chaparala H, Kahn A, Li J, Kahn L, et al. How Accurate Are We in Estimating True Stone Volume? A Comparison of Water Displacement, Ellipsoid Formula, and a CT-Based Software Tool. J Endourol. 2018;32(6):572–6. doi: 10.1089/end.2017.0937 29641351

[32] Neubauer J, Wilhelm K, Gratzke C, Bamberg F, Reisert M, Kellner E. Effect of surface-partial-volume correction and adaptive threshold on segmentation of uroliths in computed tomography. PLoS One. 2023;18(6):e0286016. doi: 10.1371/journal.pone.0286016 37352326 PMC10289361

[33] Skolarikos A, Laguna MP, Alivizatos G, Kural AR, de la Rosette JJMCH. The role for active monitoring in urinary stones: a systematic review. J Endourol. 2010;24(6):923–30. doi: 10.1089/end.2009.0670 20482232

[34] Onnis C, Virmani R, Kawai K, Nardi V, Lerman A, Cademartiri F, et al. Coronary Artery Calcification: Current Concepts and Clinical Implications. Circulation. 2024;149(3):251–66. doi: 10.1161/CIRCULATIONAHA.123.065657 38227718 PMC10794033

[35] Ko JP, Bagga B, Gozansky E, Moore WH. Solitary Pulmonary Nodule Evaluation: Pearls and Pitfalls. Semin Ultrasound CT MR. 2022;43(3):230–45. doi: 10.1053/j.sult.2022.01.006 35688534

[36] Olsen ØE. Why measure tumours? Pediatr Radiol. 2015;45(1):35–41. doi: 10.1007/s00247-014-3148-0 25552390 PMC4281379

[37] Rajendran K, Petersilka M, Henning A, Shanblatt ER, Schmidt B, Flohr TG, et al. First Clinical Photon-counting Detector CT System: Technical Evaluation. Radiology. 2022;303(1):130–8. doi: 10.1148/radiol.212579 34904876 PMC8940675

[38] Douek PC, Boccalini S, Oei EHG, Cormode DP, Pourmorteza A, Boussel L, et al. Clinical Applications of Photon-counting CT: A Review of Pioneer Studies and a Glimpse into the Future. Radiology. 2023;309(1):e222432. doi: 10.1148/radiol.222432 37787672 PMC10623209

[39] Willemink MJ, Persson M, Pourmorteza A, Pelc NJ, Fleischmann D. Photon-counting CT: Technical Principles and Clinical Prospects. Radiology. 2018;289(2):293–312. doi: 10.1148/radiol.2018172656 30179101

[40] Esquivel A, Ferrero A, Mileto A, Baffour F, Horst K, Rajiah PS, et al. Photon-Counting Detector CT: Key Points Radiologists Should Know. Korean J Radiol. 2022;23(9):854–65. doi: 10.3348/kjr.2022.0377 36047540 PMC9434736

