# Supplementary material for: Partial volume effect on kidney stones and lung nodules in CT imaging
Source: PLoS One. 2025 Oct 16;20(10):e0334597. doi: 10.1371/journal.pone.0334597 (PMC12625779; doi:10.1371/journal.pone.0334597)
Supplement: S1 Appendix — (DOCX) [file pone.0334597.s001.docx]

Appendix

Python code (voxels cut by sphere)

import random

HUI=0;HUO=-1000;CUT=-500 # Hounsfield Units Inside/Outside of sphere

for F in range(1,101): #F= sphere radius in voxel edge length

 GG=0;A1=0;AA=0;II=0;XXX=0

 for M in range(1,101): # random sphere position in the center voxel

  Ml=random.random()-0.5;M2=random.random()-0.5;M3=random.random()-0.5

  for X in range(-F,F+1): #creating voxel system in matrix

   for Y in range(-F,F+1):

    for Z in range(-F,F+1):

     A=0;I=0;D9=100000000;D10=100000000

     Q=X+Ml;W=Y+M2;E=Z+M3 #center of voxel with the following corners

     #D=vector length

Xl=Q+.5;Yl=W+.5;Z1=E+.5;Dl=(Xl*Xl+Yl*Yl+Z1*Z1)**.5

     X2=Q+.5;Y2=W+.5;Z2=E-.5;D2=(X2*X2+Y2*Y2+Z2*Z2)**.5

     X3=Q+.5;Y3=W-.5;Z3=E+.5;D3=(X3*X3+Y3*Y3+Z3*Z3)**.5

     X4=Q-.5;Y4=W+.5;Z4=E+.5;D4=(X4*X4+Y4*Y4+Z4*Z4)**.5

     X5=Q-.5;Y5=W-.5;Z5=E-.5;D5=(X5*X5+Y5*Y5+Z5*Z5)**.5

     X6=Q+.5;Y6=W-.5;Z6=E-.5;D6=(X6*X6+Y6*Y6+Z6*Z6)**.5

     X7=Q-.5;Y7=W+.5;Z7=E-.5;D7=(X7*X7+Y7*Y7+Z7*Z7)**.5

     X8=Q-.5;Y8=W-.5;Z8=E+.5;D8=(X8*X8+Y8*Y8+Z8*Z8)**.5

     if(Z==0)and(X!=0)and(Y!=0): #voxel in xy plane

       D10=((abs(Q)-.5)**2+(abs(W)-.5)**2)**.5

     if Y==0 and X!=0 and Z!=0: #voxel in xz plane

       D10=((abs(Q)-.5)**2+(abs(E)-.5)**2)**.5

     if X==0 and Y!=0 and Z!=0: #voxel in yz plane

       D10=((abs(W)-.5)**2+(abs(E)-.5)**2)**.5

     if X==0 and Y==0 and Z!=0: #voxel on z-axis

         D9=abs(E)-.5

     if Y==0 and Z==0 and X!=0: #voxel on x-axis

       D9=abs(Q)-.5

     if X==0 and Z==0 and Y!=0: #voxel on y-axis

       D9=abs(W)-.5

     if X==0 and Y==0 and Z==0: # center voxel

       D9=.5

     if Dl<F or D2<F or D3<F or D4<F or D5<F or D6<F or D7<F or D8<F or D9<F or D10<F: #corner vector smaller than sphere radius?

       I=1

     if Dl>=F or D2>=F or D3>=F or D4>=F or D5>=F or D6>=F or D7>=F or D8>=F: #corner vector larger than sphere radius?

       A=1

     if I==1 and A==1: #cut voxel

       S=0;G=0

       for XX in range(-2,3): # create 125 volume measurement points inside the voxel

         for YY in range(-2,3):

           for ZZ in range(-2,3):

             DD=((Q+XX/5)**2+(W+YY/5)**2+(E+ZZ/5)**2)**.5

             if DD<=F: # measurement point inside the sphere

               S=S+1

       G=S/125

       GG=GG+G

       AA=AA+1

       HU=G*HUI+(1-G)*HUO #average Hounsfield unit of pixel

       if HU>CUT:

         A1=A1+1

     if I==1 and A==0: #voxel completely inside sphere

       II=II+1

     XXX=XXX+1 #number of tests
